# General practice *vs* surgical-based follow-up for patients with colon cancer: randomised controlled trial

**DOI:** 10.1038/sj.bjc.6603052

**Published:** 2006-04-04

**Authors:** D A Wattchow, D P Weller, A Esterman, L S Pilotto, K McGorm, Z Hammett, C Platell, C Silagy

**Affiliations:** 1 Department of Surgery, Flinders University, Adelaide, 5042 South Australia; 2 Division of Community Health Sciences – General Practice, University of Edinburgh, 20 West Richmond St, Edinburgh, Scotland EH8 9DX, UK; 3 Division of Health Sciences, University of South Australia, Adelaide, 5000, South Australia; 4 Department of General Practice and Flinders Centre for Epidemiology & Biostatistics, Flinders University, Adelaide, 5042 South Australia; 5 Department of Surgery, Fremantle Hospital, Fremantle, 6160 Western Australia

**Keywords:** colon cancer, follow-up, general practice, surgery, investigations, quality of life

## Abstract

This trial examined the optimal setting for follow-up of patients after treatment for colon cancer by either general practitioners or surgeons. In all, 203 consenting patients who had undergone potentially curative treatment for colon cancer were randomised to follow-up by general practitioners or surgeons. Follow-up guidance recommended three monthly clinical review and annual faecal occult blood tests (FOBT) and were identical in both study arms. Primary outcome measures (measured at baseline, 12 and 24 months were (1) quality of life, SF-12; physical and mental component scores, (2) anxiety and depression: Hospital Anxiety and Depression Scale and (3) patient satisfaction: Patient Visit-Specific Questionnaire. Secondary outcomes (at 24 months) were: investigations, number and timing of recurrences and deaths. In all, 170 patients were available for follow-up at 12 months and 157 at 24 months. At 12 and 24 months there were no differences in scores for quality of life (physical component score, *P*=0.88 at 12 months; *P*=0.28 at 24 months: mental component score, *P*=0.51, *P*=0.47; adjusted), anxiety (*P*=0.72; *P*=0.11) depression (*P*=0.28; *P*=0.80) or patient satisfaction (*P*=0.06, 24 months). General practitioners ordered more FOBTs than surgeons (rate ratio 2.4, 95% CI 1.4–4.4), whereas more colonoscopies (rate ratio 0.7, 95% CI 0.5–1.0), and ultrasounds (rate ratio 0.5, 95% CI 0.3–1.0) were undertaken in the surgeon-led group. Results suggest similar recurrence, time to detection and death rates in each group. Colon cancer patients with follow-up led by surgeons or general practitioners experience similar outcomes, although patterns of investigation vary.

Colon cancer is the most common internal malignancy in Australia and other western countries, and is usually treated surgically, with or without chemotherapy. Currently follow-up is chiefly conducted in surgical clinics, aiming to detect treatable recurrent disease and provide reassurance to patients. Recurrent disease may be detected through symptoms or signs (most commonly), tests such as carcino-embryonic antigen (CEA) or faecal occult blood test (FOBT), radiological evidence or colonoscopy (Sugrue *et al*, 1991; Schoemaker *et al*, 1998; Berman *et al*, 2000; McFall *et al*, 2003; Skaife *et al*, 2003). Clinical practice guidelines in Australia and the UK highlight gaps in evidence and recommend colonoscopy every 3 to 5 years; Australian guidance includes annual FOBT (NHMRC, 1999; NICE, 2004).

Despite widespread interest in moving the focus of cancer follow-up from secondary to primary care, where evidence from other cancers suggests that it can produce equal or improved outcomes with potential economic savings (Grunfeld *et al*, 1996), there is a lack of evidence examining the setting for postoperative colon cancer follow-up.

Conversely, systematic analyses of randomised trials suggest a survival benefit from intensive *vs* nonintensive follow-up (although they provide no clear guidance on the best combination and frequency of visits or investigations) (Bruinvels *et al*, 1994; Kievit, 2002; Renehan *et al*, 2002; Northover, 2003; Pfister *et al*, 2004; Jeffery *et al*, 2006). However, pooling of data from these studies is challenging and the conclusions of meta-analyses have been questioned (Renehan *et al*, 2005) – study designs vary widely in their follow-up patterns, to the extent that some study arms deemed as ‘intensive’, have the same regimen as ‘standard’ in others.

Further, outcomes beyond recurrence (such as quality of life) have infrequently been included in these studies. While a focus on recurrence, survival and mortality may be justified in trials comparing follow-up protocols aimed at detecting recurrences, there is a strong argument that studies comparing site of follow-up (in which protocol adherence is not enforced) should logically focus on ‘process of care’ outcomes which can be linked in a logical way to setting: indeed, there have been calls for more trials in which the focus shifts from early detection of recurrences towards quality assessment and patient support (Kievit, 2002). Hence, setting of follow-up may impact on patient well-being and satisfaction with care; we hypothesise that while secondary care may provide rapid access to expertise and investigations, follow-up in general practice (GP) may lead to improvements in these outcomes.

In response to the identified lack of evidence examining setting of cancer follow-up, we report a randomised controlled trial comparing outcomes in GP-led and surgeon-led follow-up arms in patients treated for colorectal cancer. The objective of the study was to determine whether, among these patients, the setting of follow-up impacts on our primary outcomes: quality of life, psychological well-being and satisfaction with care. Also, recorded were the follow-up procedures undertaken in the two arms, and data on recurrences and death.

## METHODS

### Study design

The study was a multicentre, randomised controlled trial, which recruited patients from hospitals in South Australia, Victoria, Western Australia and Northern Territory. Potentially eligible patients were identified by direct referral from participating surgical staff, theatre and histopathology reports, cancer registries and outpatient lists. Recruitment began in March 1998 and ceased March 2001. Two-year follow up for the study sample was completed in April 2003. Ethical approval was obtained from the all institutional ethics committees of participating centres. Approval was also gained from the Ethics Committee of the Australian Institute of Health and Welfare. Participant consent included permission to obtain data on deaths/recurrences from state and national databases.

*Inclusion criteria*

- Surgery for colon cancer (including rectosigmoid) with histological grade Dukes stage A, B or C (cases of disseminated cancer were excluded).
- Completion of postsurgical chemotherapy (principally Dukes Stage C patients).
- Follow-up by GPs and surgeons available.
- Able to provide informed consent.

*Exclusion criteria*

- Rectal tumours (current practice for rectal cancer follow-up requires regular sigmoidoscopy which would not be undertaken by many GPs).
- Significant polyps discovered at initial colonoscopy (or at subsequent completion colonoscopy) that indicated increased frequency of colonoscopic monitoring.
- Any other condition that warranted increased intensity of surveillance with respect of colon cancer follow-up.

Following agreement from the treating surgeon and GP, eligible patients were invited to participate in the study at their final postsurgical follow-up visit (4–6 weeks after surgery or completion of postsurgical chemotherapy). Consenting patients were then randomly allocated to either ‘GP-led’ or ‘surgeon-led’ follow-up using an Excel random number generator. Randomisation was conducted by the study researchers, who were not involved in the design of the study or the clinical care of the patients, and was concealed until the interventions were assigned. The study was single-blinded. Researchers at all times were unaware of the patient allocation until after the randomisation process. Patients were reviewed by GPs in their practice rooms and surgeons in their surgical clinics.

### Interventions

The setting and environment of follow-up (primary *vs* secondary care) constituted our intervention. Follow-up guidance, based on current clinical practice and guidance (Table 1) was provided, and inserted into either the patient's GP or surgeon/hospital records. Nevertheless, in accordance with the study's pragmatic design, there was no compulsion for clinicians in either setting to adhere to the guidance. Participating clinicians received regular study information from contact with the study researcher and a newsletter. Patients allocated to ‘GP-led’ follow-up could be referred back to surgical clinics at any point in the study; similarly, patients in the ‘surgeon-led’ follow-up group could consult their GP at any time during the course of the study.

### Outcome measurement

The study used validated outcome measures; self-completion questionnaires which included a range of validated instruments (below) were developed and piloted among colorectal cancer patients treated at Flinders Medical Centre. Instruments were provided directly to patients in clinic settings or sent by post. Clinical data were obtained through GP and hospital case note audit, using a standardised data extraction sheet at 12 and 24 months postrecruitment. Cancer incidence and deaths were determined through data linkage with state-based cancer registries and the National Death Index (AIHW, 2004).

#### Primary outcomes

*Measured at baseline, 12 and 24 months*

- quality of life based on SF-12 Physical (PCS) and mental health component (MCS) scores (Ware *et al*, 1995),
- depression and anxiety based on the Hospital Anxiety and Depression Scale (HADS) (Zigmond and Snaith, 1983).

*Measured at 24 months only:*

- Satisfaction based on the Patient Visit-Specific Questionnaire (PSVQ) (Davies and Ware, 1991).

#### Secondary outcomes

*Measured at 24 months only:*

- the number and type of investigations (blood tests, FOBT, colonoscopies and radiological investigations),
- number and time to detection of recurrences,
- deaths from all causes at 2 years postentry into the study.

### Sample size and statistical analysis

All power calculations were undertaken using a power of 80% with a 0.05 two-sided significance level. Power calculations were based on our primary outcome measures.

*SF-12*: A sample size of 64 in each group was required to detect a difference in mean PCS or MCS score of 5 at 24 months, assuming a standard deviation of 10, using a two group *t*-test.

*HADS*: A sample size of 64 in each group was required to detect a difference in mean HADS Anxiety or Depression score of 1.5 at 24 months, assuming a standard deviation of 3.0, and using a two group *t*-test.

*PSVQ*: A sample size of 62 in each group was required to detect a difference of 20% in the proportion rating an item as ‘excellent’ or ‘very good’ 24 months (90% in one group compared with 70% in the other).

Allowing for a dropout rate of 25%, we set our recruitment target at 100 patients in each treatment arm. The study was not powered to measure differences in recurrence and mortality data at 24 months follow-up. These data are nevertheless analysed and reported, and recording of recurrence and mortality in study participants is on going.

Unadjusted comparisons between the two treatment arms for the HADS and SF-12 scales at 12 and 24 months were undertaken using Exact Mann–Whitney *U*-tests. Comparisons adjusting for baseline values were undertaken using analysis of covariance on ranks. The percentage satisfied or very satisfied on PSVQ items were compared in the two treatment arms using Fisher's exact tests at 12 and 24 months. Rates of investigations undertaken, recurrences and deaths per months on trial were compared using Fisher's exact tests. Time to recurrence and death were analysed using a Kaplan–Meier analysis with Log rank test. Trial participants and patients in the SA Cancer Registry were compared using *χ*^2^-tests. Although randomisation was by individual, there was concern that results might be clustered by hospital or surgeon. For the counts of investigations a random effects Poisson regression model was fitted to establish whether or not observations could be considered independent by comparing with a usual Poisson regression model. The analysis showed virtually no difference between the models and therefore observations were considered to be independent.

Double data-entry was used and analysis was blinded, on an intention-to-treat basis.

## RESULTS

### Participant flow

**Figure 1:**
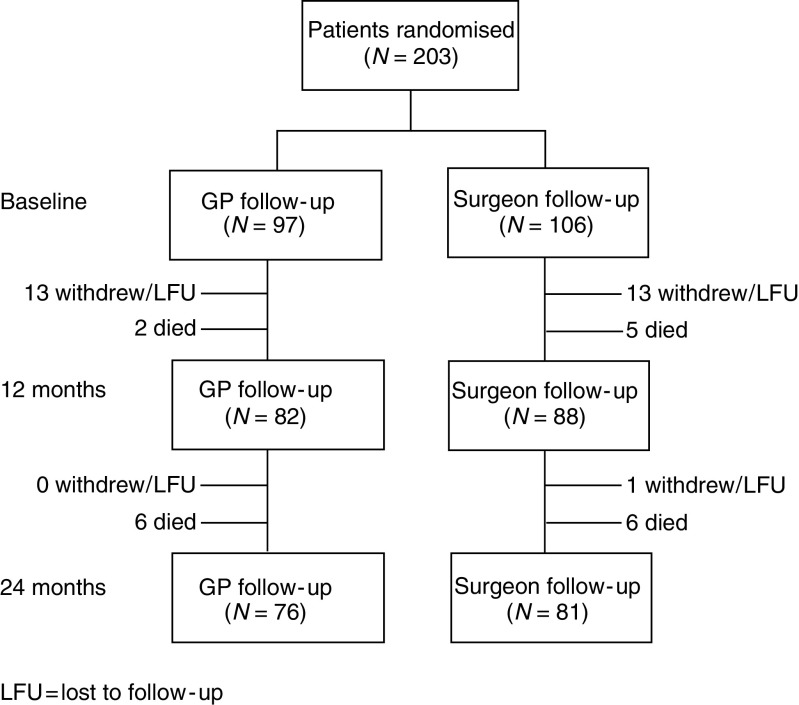
Patient flow through study.

Figure 1 shows the flow of participants through the study. Of the 611 patients assessed, 340 were eligible, and 203 agreed to randomisation. The main reason for patients declining participation (137) was a desire for the choice over setting for follow-up to be made either by themselves or their surgeon. Withdrawal was viewed as noncompletion of questionnaires (primary outcome measures) – data on deaths were still collected. Reasons given for withdrawing were participant commitment (10), concern over the time involved (4), lack of understanding of the study (1) and one did not ‘wish to be reminded of their illness’. The remaining patients gave no explanation, but the withdrawals were equally distributed between the groups. There were 76 patients in the GP group, and 81 in the surgical group after 24 months of follow-up, meeting the numbers required for statistical validity. Analysis was on an ‘intention to treat’ basis.

Table 2 shows the characteristics of the trial participants at baseline. Of patients, 70% were recruited in SA. Groups had similar characteristics with the exception of education, where there was a trend towards higher levels of education in the surgeon follow-up group. To examine external validity of our sample we compared age, sex and Dukes staging with SA Cancer Registry data (Cancer Council of SA, 2001) (included in Table 2) using *χ*^2^-tests. Study participants did not differ significantly compared with registry patients with respect to gender (*P*=0.53) and Dukes staging (*P*=0.12), but had a slightly narrower age distribution (*P*=0.05).

### Depression and anxiety (Table 3)

A HADS score of 8–10 is borderline and 11–21 abnormal. There were no statistically significant differences between the two groups at either 12 or 24 months follow-up and, on average, study participants were in the normal range at all times.

### Quality of life (Table 3)

A score of 50 on the SF12 scale is normal for the Australian population (a difference of 10 points is clinically significant). Patients in our study had reduced subjective quality of life with respect to physical health at baseline and this improved as the study progressed. Comparison of scores in the two arms of the study revealed no statistically significant differences after 12 or 24 months of follow-up.

### Patient satisfaction (Table 4)

There were no statistically significant differences between study groups in the percentage of patients rating each item ‘excellent’ or ‘very good’ on the PSVQ scale, administered at 24 months, and with the exception of ‘contacting the doctor by telephone’ and ‘time spent in the waiting room’, the combined study group reported high levels of satisfaction with their care.

### Follow-up visits and investigations (Table 5)

Patients in both groups were expected to visit their treating clinician for follow-up on a quarterly basis. In the GP follow-up group, patients visited their GP on average 1.27 times per quarter (718 visits over 565 patient–months on trial). Those in the surgeon follow-up group visited the surgeon on average 0.84 times per quarter (457 visits over 543 patient–months on trial).

Patients in the GP-led follow-up arm were significantly more likely to have one or more FOBTs, whereas patients in the surgeon-led follow-up arm were significantly more likely to have one or more ultrasounds, and one or more colonoscopies. We recorded, during the study period, the numbers of colonoscopies performed in response to development of symptoms (change in bowel habit or bleeding) or positive FOBT. Such indications existed in 32 patients in the GP group and 38 patients in the surgical group (Fisher's exact test *P*=0.30).

### Recurrence and death

The study was not powered to detect differences in death or recurrence rates; nevertheless, no clear trends emerged. The recurrence rate in the GP follow-up group was 7.1 per 1000 months on trial compared with 8.0 per 1000 months on trial in the surgeon follow-up group (Fisher's exact test *P*=0.92). For those with a recurrence, the median time to detection was 9.5 months from recruitment for the GP follow-up group, and 8.0 months for the surgeon follow-up group. A Kaplan–Meier survival analysis was undertaken and a Log rank test found there to be no difference in time to recurrence between study groups (*P*=0.76).

The death rate in the GP follow-up group was 6.6 per 1000 months of follow-up compared with 5.4 per 1000 months of follow-up in the surgeon follow-up group (Fisher's exact test *P*=0.67). For those with who died, the median time to death was 31 months for the GP follow-up group, and 20 months for the surgeon follow-up group. A Kaplan–Meier survival analysis was again undertaken and a Log rank test found there to be no significant difference in time to death between study groups (*P*=0.69).

## DISCUSSION

We found no significant differences between postsurgical colon cancer patients undergoing either GP-led or surgeon-led follow-up for our primary outcome measures of quality of life, depression and anxiety and patient satisfaction. There were insufficient numbers to demonstrate differences in number of recurrences and deaths, but the data do not suggest the emergence of any trend, and these outcomes will be examined in longer-term follow-up of our study participants. Although equivalent follow-up guidance was provided to those involved in the care of study participants there was a higher rate of FOB testing in the GP-led follow-up arm and higher numbers of colonoscopies and ultrasounds in the surgeon-led arm.

As participants were typical of a western population with a high incidence of colorectal cancer, the findings of the study are generalisable to other countries with well-developed systems of primary care and surgical services.

By issuing identical follow-up guidance to both study arms, our study sought a best estimate of the effect of setting. It was pragmatic in design; blinding of trial participants was not possible after randomisation. Further, there was inevitably a degree of crossover between the two arms of our study – it was neither possible nor desirable to limit access to GPs in patients allocated to the surgical arm of the study or vica versa. However, participants largely adhered to their allocated follow-up groups. The study relied on self-completion questionnaires and case-note audit for much of its data, involving a significant response burden, but numbers of questionnaires with missing data were low at all stages of follow-up. GP and hospital case-notes are not always complete, and missing data can distort the results of audit-based studies (Wilson *et al*, 2000; van Walraven and Demers, 2001). Nevertheless, the information we required from case notes was quite straightforward, and generally very accessible. Further, the standardised data extraction sheets we developed as part of the study helped ensure accuracy and consistency.

Our study's finding are consistent with previous research comparing GP *vs* hospital follow-up for breast cancer patients (Grunfeld *et al*, 1996). They also complement previous studies and systematic reviews which have examined the intensity of follow-up regimens for cancers of the large bowel; studies attributing increased survival to ‘intensive’ follow-up reveal that a substantial portion of this increase is due to factors other than detection of treatable disease (Renehan *et al*, 2005). As comorbid illnesses are present in the majority of patients with colorectal cancer (Payne and Meyer, 1995) this may additionally point to the value of GP-based follow-up.

While many studies have focused on the detection of treatable disease in the follow-up period our findings provide evidence regarding patients' quality of life and satisfaction with care, the importance of which has been widely recognised. This study challenges follow-up practices for colon cancer which have traditionally, in countries such as Australia and the UK, been led by secondary care. National guidance in these and similar countries has had little evidence upon which to base recommendations for setting of follow-up – although UK guidance includes the use of multidisciplinary teams.

Primary care has a growing role in cancer management (Campbell *et al*, 2002). Our conclusions may be strengthened in the UK by a study of hospital *vs* GP follow-up, although the results of this study will not be available for some time, and it differs inasmuch as diagnostic protocols are not held constant in the two arms of the study (Primrose *et al*, 2005). At present there is little evidence to guide the development of follow-up protocols for common cancers such as prostate, bowel and lung cancer; further research on cost-effectiveness of various follow-up protocols is required, with a careful analysis of the potential and limitations of primary care. Capacity and workforce issues are of particular importance in developing recommendations involving transfer of follow-up from surgical to GP settings, and should be incorporated in future research.

## Figures and Tables

**Table 1:** Recommended follow-up regimen (5 years in total)

*The patient should be reviewed*
1. Three monthly for the first 2 years postoperatively
2. Then 6 monthly for the next 3 years

*Patient history*
Please ask the following (or similar) questions to your patient
1. What is your bowel habit? Has there been any change lately?
2. Have you noticed any bleeding in the stools or from the anus?
3. Have you experienced any abdominal pains of more than a few days' duration?
4. Have you experienced any other pains, for example in your back, chest or legs?
5. Have you noticed any weight loss?
6. Have you been feeling tired or lethargic?

*Physical examination*
Assess the patient for
1. Colour
2. Enlarged neck nodes
3. Abdominal masses, for example, the liver, wound deposits or ascites

*Diagnostic tests*
Recent studies have raised doubts as to the value of many diagnostic tests in the detection of recurrent or metastatic disease. However, there is value in performing.
1. Annual FOBT (faecal occult blood test)
2. A colonoscopy every 3 years

*Note*: If the patient experiences any positive signs or symptoms, they should be appropriately investigated. Please use the case note provided for each relevant follow-up consultation.

**Table 2:** Characteristics of colon cancer patients: Study+South Australian population

	**Study participants (*n*=203)**						**South Australian Cancer registry 1995 to 2002**		
	**GP**		**Surgeon**		**Total**				
	***n***	**%**	***n***	**%**	***n***	**%**		***n***	**%**
*State*									
SA	73	75.3	69	65.1	142	70.0			
NT	0	.0	1	.9	1	.5			
WA	10	10.3	21	19.8	31	15.3			
VIC	14	14.4	15	14.2	29	14.3			
									
*Gender*									
Male	60	61.9	57	53.8	117	57.6	Male	786	51.1
Female	37	38.1	49	46.2	86	42.4	Female	752	48.9
Total	97	100.0	106	100.0	203	100.0	Total	1538	100.0
									
*Age group*									
<60	10	10.3	16	15.1	26	12.8	<60	258	16.8
60–69	18	18.6	28	26.4	46	22.7	60–69	368	23.9
70–79	47	48.5	46	43.4	93	45.8	70–79	555	36.1
80+	22	22.7	16	15.1	38	18.7	80+	357	23.2
Total	97	100.0	106	100.0	203	100.0	Total	1538	100.0
									
*Education*									
Primary only	26	27.7	16	15.7	42	21.4			
Secondary	28	29.8	19	18.6	47	24.0			
School leaver	16	17.0	26	25.5	42	21.4			
Postsecondary	8	8.5	23	22.5	31	15.8			
Unknown	16	17.0	18	17.6	34	17.3			
Total	94	100.0	102	100.0	196	100.0			
									
*Dukes stage*									
A	24	24.7	23	21.7	47	23.2	A	273	17.7
B	43	44.3	53	50.0	96	47.3	B	733	47.6
C	30	30.9	30	28.3	60	29.6	C	532	34.6
Total	97	100.0	106	100.0	203	100.0	Total	1538	100.0

**Table 3:** HADS and SF-12 scores

	**GP**			**Surgeon**			**Significance**	
**Outcome**	*n* *	**median**	**IQR**	*n* *	**median**	**IQR**	**unadjusted^a^**	**adjusted^b^**
*HADS anxiety*								
Baseline	95	4.0	5.0	106	4.0	4.0		
12 months	81	4.0	3.5	87	4.0	4.0	0.932	0.716
24 months	76	4.0	5.0	81	5.0	4.5	0.440	0.106
								
*HADS depression*								
Baseline	97	4.0	5.0	106	3.0	4.0		
12 months	81	4.0	4.0	87	4.0	5.0	0.213	0.283
24 months	76	4.0	5.0	81	3.0	4.0	0.540	0.796
								
*SF-12 PCS*								
Baseline	95	42.3	20.5	105	40.5	19.0		
12 months	82	49.3	11.2	88	55.9	14.3	0.576	0.887
24 months	76	48.5	17.7	79	50.4	14.4	0.194	0.279
								
*SF-12 MCS*								
Baseline	95	55.0	12.9	105	55.1	14.5		
12 months	81	49.9	13.0	88	55.6	10.8	0.558	0.510
24 months	74	54.4	11.8	79	55.9	11.2	0.448	0.474

* Not all participants identified as ‘available for follow-up’ (Figure 1) completed every instrument.

a Mann–Whitney *U*-test.

b Adjusted for baseline value using analysis of covariance on ranks.

**Table 4:** PSVQ at 24 months follow-up – those rating items as ‘excellent’ or ‘very good’

	**GP (*n*=76)**		**Surgeon (*n*=81)**		
**PSVQ Item**	***n***	**%**	***n***	**%**	**Sig.^a^**
How long they waits for an appointment	60	82.2	60	82.2	0.690
Convenience of location	61	83.6	57	78.1	0.179
Getting through to Doctor by phone	50	68.5	45	61.6	0.257
Time in waiting room to see Doctor	41	56.2	43	58.9	1.000
Average time spent with Doctor	56	76.7	50	68.5	0.116
Explanations of your condition and what has been done for you	61	83.6	59	80.8	0.326
Technical skills of Doctor	65	89.0	66	90.4	0.496
Personal manner of Doctor	71	97.3	69	94.5	0.081
Overall satisfaction with the care from your Doctor	70	95.9	67	91.8	0.064

a Fisher's exact test.

**Table 5:** Number of patients with one or more of the following pathology and diagnostic tests undertaken per 1000 months on trial

	**GP (*n*=97)**		**Surgeon (*n*=106)**				
**Test**	**Number**	**Rate**	**Number**	**Rate**	**Rate ratio**	**95% CI for rate ratio**	**sig.^a^**
FOBT	46	23.6	16	9.8	2.4	1.4–4.4	0.003
CEA	33	19.5	48	29.5	0.7	0.4–1.0	0.083
CBP	52	30.7	50	30.7	1.0	0.7–1.5	1.000
LFT	56	33.0	47	28.8	1.1	0.8–1.7	0.556
Xray	30	17.7	20	12.3	1.4	0.8–2.7	0.256
Ultrasound	16	9.4	30	18.4	0.5	0.3–1.0	0.040
CT scan	26	15.3	34	20.9	0.7	0.4–1.3	0.291
Colonoscopy	55	32.4	79	48.5	0.7	0.5–1.0	0.027
Endoscopy	6	3.5	10	6.1	0.6	0.2–1.8	0.408
Sigmoidoscopy	5	2.9	15	8.6	0.3	0.1–1.0	0.051
DRE	19	11.2	28	17.2	0.7	0.3–1.2	0.193

a Fisher's exact test.
